# Sensor-based assessment of the acute effects of NMES combined with weighted squats on muscle activation and lower limb joint coordination during the stop-and-cut task

**DOI:** 10.3389/fphys.2026.1843577

**Published:** 2026-07-01

**Authors:** Xiaotian Guo, Qinglin Wang

**Affiliations:** Physical Education College, South-Central Minzu University, Wuhan, China

**Keywords:** joint coordination, muscle activation, neuromuscular electrical stimulation, stop-and-cut, weighted squat

## Abstract

**Background:**

The stop-and-cut (SAC) is a key movement pattern in many competitive sports and directly influences braking, directional transition, and re-acceleration performance. However, the acute effects of neuromuscular electrical stimulation (NMES) combined with weighted squats on SAC-related performance, muscle activation, and lower limb joint coordination remain unclear.

**Methods:**

Thirty-six male basketball athletes were randomly assigned to a loaded squat group (LS), a sham stimulation combined with loaded squat group (SSLS), or a true stimulation combined with loaded squat group (TSLS). A randomized, single-blind, pre–post intervention design was adopted. The key feature of this study was the integration of external performance testing with synchronized motion-sensor assessment during the SAC task, including surface electromyography, infrared 3D motion capture, and force plate data. V-cut test performance was used as the external indicator, while root mean square (RMS), integrated electromyography (iEMG), and lower limb joint coordination variables were extracted during the SAC support phase. A 3-group × 2-time mixed-design ANOVA was used for statistical analysis.

**Results:**

Only the TSLS group showed a significant improvement in V-cut test performance, with a significant intervention effect observed (F = 16.27, P< 0.001, η² = 0.606). During the SAC full support phase, significant group × time interactions in RMS were found for GM (F = 14.62, P< 0.001, η² = 0.587), VM (F = 13.48, P< 0.001, η² = 0.565), and BF (F = 12.91, P< 0.001, η² = 0.551). Similar significant interactions in iEMG were also observed for GM (F = 15.26, P< 0.001, η² = 0.601), VM (F = 14.11, P< 0.001, η² = 0.578), and BF (F = 13.37, P< 0.001, η² = 0.562). For lower limb joint coordination, more evident significant effects were observed in the hip–knee and knee–ankle couplings, whereas fewer significant changes were found in the hip–ankle coupling.

**Conclusion:**

NMES combined with weighted squats was more effective than weighted squats alone or sham stimulation combined with weighted squats in acutely improving SAC-related performance. This improvement was accompanied by enhanced activation of key lower limb muscles and more evident changes in lower limb joint coordination, suggesting that this combined intervention may be an effective acute strategy for improving SAC-related movement performance and control.

## 1 Introduction

In competitive sports such as basketball, soccer, and handball, athletes frequently perform rapid deceleration, directional transition, lateral cutting, and re-acceleration tasks. Among these actions, the stop-and-cut (SAC) is one of the most common and critical fundamental movement patterns (Oliver et al., 2024). SAC is widely involved in dribbling penetration, evasive maneuvers, defensive slides, and offensive-to-defensive transitions, and it directly influences an athlete’s ability to brake, control posture, change direction, and execute the next movement at high speed (Aztarain-Cardiel et al., 2024). Because SAC is typically performed under conditions of high intensity, short duration, and substantial task complexity, the quality of its execution has an important influence on sport-specific movement efficiency and overall athletic performance (Georgoulis et al., 2023). Therefore, for sports requiring rapid changes of direction, SAC ability is regarded as a key indicator of competitive performance.

The execution of SAC depends not only on lower-limb force production, but also on precise neuromuscular regulation and inter-joint coordination. During the support phase, athletes must complete braking, body stabilization, and preparation for re-acceleration within a very short time window. Lower-limb joint coordination was selected as a core outcome because SAC performance depends not only on the magnitude of joint movements or forces, but also on how adjacent joints interact to control braking, weight acceptance, postural stability, and propulsion within a very short support phase. While kinematic or kinetic variables can quantify joint angles, velocities, or ground reaction forces individually, they do not capture the dynamic interplay between joints that determines movement efficiency and control. By analyzing joint coordination patterns, we can assess whether the intervention alters how the hip, knee, and ankle work together to achieve stable and effective directional changes. This approach provides a mechanistic perspective on how NMES combined with weighted squats may acutely influence the control strategies underlying SAC, complementing traditional kinematic and kinetic analyses. Accordingly, the activation of key lower-limb muscles, including the gluteal muscles, quadriceps, hamstrings, and calf-related muscles, directly affects movement efficiency and control (Liu et al., 2024). In parallel, the coordination among the hip, knee, and ankle joints determines how impact forces are absorbed, transferred, and re-generated during directional transition (Thurlow et al., 2024). Previous studies have shown that surface electromyography (EMG) variables, such as root mean square (RMS) and integrated electromyography (iEMG), can effectively reflect instantaneous muscle activation intensity and overall electrical activity, whereas joint coordination metrics can reveal the dynamic control relationship among lower-limb joints during movement (Keiner et al., 2022). Therefore, a comprehensive understanding of SAC requires simultaneous attention to both muscle activation characteristics and joint coordination behavior.

Neuromuscular electrical stimulation (NMES) is a commonly used intervention strategy that induces neuromuscular excitation through externally applied electrical currents, thereby enhancing motor unit recruitment and neural drive. In recent years, NMES has been increasingly used in athletic training, pre-competition preparation, and sports rehabilitation (Li et al., 2025). Previous research has shown that properly designed conditioning activities can acutely improve explosive and change-of-direction performance, and that combining NMES with active resistance exercise may induce stronger acute enhancement effects than traditional resistance exercise alone. Weighted squats are widely used as a conditioning activity because they provide substantial mechanical loading; however, their acute transfer effect may be limited for sport-specific tasks such as SAC, which require rapid braking and re-acceleration within a constrained support period (Maffiuletti et al., 2023). When NMES is superimposed on weighted squats, the combined mechanical and electrical stimuli may theoretically increase neuromuscular excitation and facilitate the recruitment of lower-limb muscles involved in braking, stabilization, and re-acceleration. Based on this rationale, NMES combined with weighted squats may be considered a potentially relevant conditioning activity before SAC performance. Because the present study focused on acute effects, the time-dependent nature of post-activation performance enhancement (PAPE) should be considered. PAPE responses typically occur within minutes after a conditioning activity and reflect the balance between performance-enhancing potentiation and performance-impairing fatigue. If the post-conditioning interval is too short, residual fatigue may mask the potentiation effect; if the interval is too long, the potentiation effect may gradually decline (Li et al., 2023a). Therefore, the acute response is not a stable or long-lasting adaptation, but a transient neuromuscular state that depends on the timing of post-intervention assessment. In this context, an acute intervention framework is particularly relevant for sport-specific preparation because athletes often use short-term conditioning activities during warm-up or pre-competition routines to optimize immediate performance. Examining the acute effects of NMES combined with weighted squats before SAC is scientifically important because it helps clarify whether this combined strategy can induce a short-term neuromuscular state that is favorable for rapid braking, stabilization, and re-acceleration, rather than only producing general training adaptations over repeated sessions.

Therefore, the scientific gap addressed in the present study is not the use of synchronized EMG, motion capture, and force plate measurements per se, as these methods are well established in sports biomechanics. Rather, the gap lies in the limited evidence on how NMES combined with weighted squats acutely affects SAC-related performance and whether any performance change is accompanied by specific changes in muscle activation and lower-limb joint coordination. In this study, synchronized EMG, three-dimensional motion capture, and force plate data were used as complementary assessment tools to link external performance outcomes with neuromuscular and coordinative responses during the SAC task (Li et al., 2023b). Compared with assessments based only on external test outcomes, sensor-based biomechanical evaluation can more objectively reveal the internal neuromuscular and coordinative mechanisms associated with SAC execution.

For sport-specific movements such as SAC, the value of sensor application is particularly prominent. SAC includes rapid braking, weight acceptance, postural adjustment, and propulsion within a very short support phase, making it difficult to fully characterize with single-indicator testing alone. A multi-sensor approach makes it possible to link external movement outcomes with internal control processes, thereby identifying how acute interventions alter muscle recruitment and lower-limb coordination during task execution. From the perspective of sports sensor applications, such a framework is not only useful for mechanism-oriented biomechanical research, but also has practical value for performance monitoring, training optimization, and evidence-based intervention evaluation in athletes. From a task-specific perspective, weighted squats combined with NMES may be particularly suitable as a conditioning activity before the SAC task. The SAC requires athletes to rapidly decelerate, stabilize the body, control frontal- and sagittal-plane lower-limb motion, and subsequently re-accelerate in a new direction within a very short support period. These requirements depend heavily on the force-generating and stabilizing functions of the hip and knee extensors, as well as the coordinated contribution of the posterior thigh muscles. Weighted squats provide a high-load, multi-joint stimulus involving the hip, knee, and ankle joints, which is biomechanically closer to the lower-limb extension and force-absorption demands of SAC than isolated or less task-specific conditioning exercises. When NMES is superimposed on weighted squats, the external electrical stimulus may further increase the recruitment of motor units in key muscles such as the gluteus maximus and rectus femoris during the conditioning activity, thereby enhancing neuromuscular readiness for the subsequent braking and re-acceleration phases of SAC.

Against this background, the present study aimed to examine whether NMES combined with weighted squats produces greater acute improvements in SAC-related performance than weighted squats alone or sham stimulation combined with weighted squats, and to determine whether such performance changes are accompanied by specific neuromuscular and inter-joint coordination responses. The significance of this study lies not only in testing an acute conditioning strategy for improving change-of-direction performance, but also in linking external performance outcomes with internal movement-control mechanisms during a basketball-relevant SAC task. Although previous studies have reported acute performance enhancement after conditioning activities, limited evidence has explained how NMES combined with weighted squats affects both muscle activation and lower-limb coordination during a sport-specific directional-change task. Therefore, the novelty of this study is that it integrates V-cut performance, EMG-derived muscle activation variables, and sagittal-plane inter-joint coordination analysis to clarify whether the combined intervention induces a task-specific neuromuscular response rather than only a general performance change. This approach may provide practical evidence for designing pre-competition conditioning strategies and biomechanical evidence for understanding how acute neuromuscular interventions influence SAC movement control in basketball athletes.

## 2 Participants and methods

### 2.1 Participants

This study employed convenience sampling to recruit male basketball-specific athletes as participants. The primary reason for selecting this specific group was that basketball frequently involves movements such as sudden stops, lateral cuts, changes of direction, and re-acceleration. As the participants had undergone long-term specialized training related to the stop-and-cut (SAC), they were able to perform the SAC tasks involved in this study in a stable and standardized manner. Therefore, they are well-suited to serve as a representative sample for investigating SAC movement performance and its neuromuscular control characteristics. All participants were right-handed with a dominant right lower limb and possessed a stable foundation in basketball-specific training. They were competitive male basketball athletes who had obtained at least the Chinese National Level 2 athlete certification or an equivalent competitive qualification. All participants had participated in systematic basketball training for at least three years and were currently engaged in regular basketball-specific training no fewer than four sessions per week. Their routine training included sprinting, deceleration, change-of-direction drills, defensive sliding, jumping, and lower-limb resistance exercises. In addition, they were familiar with loaded squat exercises and sport-specific movements such as sudden stops, lateral cuts, and shuttle-run tasks. Therefore, the participants had sufficient competitive background and task familiarity to perform the SAC and V-cut tests in a stable and standardized manner.

Sample size estimation was performed using G*Power 3.1 software. Based on the three-group repeated-measures design of this study, with a statistical power (1−β) of 0.80, a significance level (α) of 0.05, and an effect size of f = 0.40, the calculated minimum sample size was 26 participants. The effect size of f = 0.40 was selected according to Cohen’s conventional criteria for ANOVA, in which f = 0.40 is interpreted as a large effect size. This value was used because the present study examined an acute neuromuscular conditioning intervention and, in the absence of a directly comparable prior study using the same NMES combined with weighted squat protocol during SAC, Cohen’s conventional large-effect estimate was considered appropriate for *a priori* sample size estimation (Faul et al., 2007).

To account for potential dropouts and invalid data, a total of 36 participants were ultimately included and randomly assigned to the weighted squat group, the real-stimulus combined with weighted squat group, and the sham-stimulus combined with weighted squat group, with 12 participants in each group.

Inclusion criteria were: ① Male, aged 18–25 years; ② National Level 2 or higher basketball players; ③ At least 3 years of systematic specialized basketball training experience; ④ Participating in specialized training at least 4 times per week; ⑤ Right-handed with a dominant right lower limb; ⑥ No history of severe musculoskeletal injuries in the lower limbs within the past 6 months; ⑦ Able to proficiently perform weighted squats, SAC, and related test tasks; ⑧ No history of neurological diseases or contraindications for neuromuscular electrical stimulation. Exclusion criteria: ① History of acute injury, surgery, or residual functional impairment involving the hip, knee, or ankle joints within the past 6 months; ② istory of neurological disorders, balance dysfunction, or severe lower back and leg pain; ③ Presence of a cardiac pacemaker or other implanted devices unsuitable for electrical stimulation; ④ Inability to complete the entire testing process during the experiment due to pain, significant fatigue, or other reasons; ⑤ Incomplete or invalid data collection.

All participants signed an informed consent form prior to the study and were fully informed of the experimental procedure, potential risks, and their right to withdraw. This study was conducted after approval by the institutional ethics committee. There were no statistically significant differences among the three groups in baseline characteristics such as age, height, weight, BMI, and years of training (P > 0.05), indicating good comparability between groups (**Table 1**).

**Table 1:** Comparison of participant characteristics among groups (M ± SD, N = 36).

Variable	LS	TSLS	SSLS	F value	P value
Age (years)	21.13 ± 1.36	20.88 ± 1.46	21.25 ± 1.28	0.19	0.831
Height (cm)	182.38 ± 4.27	181.75 ± 4.83	182.88 ± 4.15	0.14	0.870
Body mass (kg)	76.25 ± 6.14	75.63 ± 5.88	76.88 ± 6.02	0.10	0.905
BMI (kg/m²)	22.91 ± 1.24	22.83 ± 1.11	23.00 ± 1.19	0.05	0.954
Training experience (years)	8.13 ± 1.36	8.88 ± 1.25	8.25 ± 1.28	0.18	0.839

Between-group comparisons of baseline characteristics were performed using one-way analysis of variance (ANOVA). The results showed no statistically significant differences among the three groups in age, height, body mass, BMI, or training experience (all P > 0.05), indicating good baseline homogeneity across groups.

### 2.2 Experimental design

This study employed a randomized, single-blind, parallel-group design. Participants who met the inclusion criteria were randomly assigned to three groups: the loaded squat group (LS), the sham stimulation combined with loaded squat group (SSLS), and the true stimulation combined with loaded squat group (TSLS). In this single-blind design, the participants were blinded to the specific stimulation condition (real or sham), whereas the investigators administering the interventions were aware of the group assignments. Data analysts were also blinded to group allocation to minimize bias during post-processing and statistical analysis. The objective of this study was to compare the effects of different exercise modalities on the post-activation enhancement of the SAC, muscle activation patterns, and lower limb joint coordination and control.

This study employed a single-session acute intervention with pre- and post-test design. All participants completed a familiarization session prior to the formal experiment to become accustomed to the loaded squat movement, the neuromuscular electrical stimulation procedure, the SAC task, and the movement performance testing protocol. On the day of the formal experiment, all participants sequentially completed basic information registration and anthropometric measurements, determination of the dominant leg, application of surface electromyography electrodes, placement of reflective markers, a standardized warm-up, baseline testing, group-specific exercises, recovery, and post-test.

The standardized warm-up consisted of 5 minutes of light jogging, dynamic stretching, low-intensity bodyweight squats, and lateral movement exercises, followed by 2–3 repetitions of submaximal-intensity stop-and-go drills. After the baseline test, participants underwent the corresponding conditional activity intervention based on their random group assignment. Following the intervention, all participants underwent a 6-minute recovery period before proceeding to the post-test. The pre-test and post-test protocols were identical, both including the SAC test, the V-cut test, and the simultaneous collection of kinematic, kinetic, and surface electromyography signals.

One-Repetition Maximum Assessment: The one-repetition maximum (1RM) for the barbell back squat was assessed before the formal experimental testing session. To avoid acute fatigue affecting the main experiment, the 1RM assessment was completed during a separate familiarization and strength-testing session at least 48 hours before the formal intervention and testing session. Before the 1RM test, participants performed a standardized warm-up consisting of light jogging, dynamic lower-limb stretching, bodyweight squats, and several submaximal squat sets with progressively increasing loads. The back squat 1RM was directly tested rather than estimated. After warm-up, the load was progressively increased until the participant could complete one technically valid repetition through the required range of motion but could not complete a second repetition with proper technique. A rest interval of 3–5 minutes was provided between attempts to minimize fatigue. The highest load successfully lifted with correct technique was recorded as the individual 1RM. The 70% 1RM load used in the intervention was then calculated individually based on this measured value.

Post-Intervention Recovery Interval: A 6-min recovery period was applied after the intervention before the post-test measurements. This duration was chosen based on previous research on PAPE, which indicates that performance potentiation can emerge several minutes after a conditioning activity once acute fatigue has subsided, typically within 4–10 min post-conditioning. A 6-min interval falls within this commonly used time window and has been shown in prior studies to allow the balance between potentiation and fatigue to favor performance enhancement in explosive and change-of-direction tasks (Wong et al., 2012). Therefore, a 6-min recovery period was considered appropriate to capture potential acute effects of the NMES combined with weighted squat intervention on SAC-related performance.

To minimize the risk of excessive muscular fatigue during the combined NMES and weighted squat intervention, several strategies were implemented. First, the load was set at 70% of 1RM, which is commonly used for PAPE without inducing substantial fatigue. Each set included only three repetitions, and stimulation phases were interspersed with 12-second rest intervals, with a 2-minute rest between sets to allow partial recovery. Participants were closely monitored for signs of fatigue, and the intervention was designed as an acute, single-session protocol to prevent cumulative fatigue effects. Previous studies indicate that NMES combined with submaximal resistance exercise at similar intensities can enhance neuromuscular activation while maintaining tolerable fatigue levels in trained athletes. These considerations ensured that the conditioning activity augmented neuromuscular readiness without imposing excessive fatigue that could confound SAC or V-cut performance measurements.

### 2.3 Stimulation intervention protocols

#### 2.3.1 LS (loaded squat group)

Based on previous studies, the load was set at 70% of 1RM, a commonly used load for PAPE. Participants performed barbell back squats at 70% of 1RM, completing 3 sets of 3 repetitions each with a 2-minute rest between sets (Fernández-Elías et al., 2022).

#### 2.3.2 TSLS

Participants performed squats with a 70% 1RM load while receiving neuromuscular electrical stimulation. The stimulation sites were the bilateral rectus femoris and gluteus maximus muscles. Stimulation parameters were as follows: frequency 75 Hz, pulse width 400 μs, stimulation duration 6 s, interval 12 s, and stimulation intensity 90% of the individual’s maximum tolerable threshold. During each 6-second stimulation phase, participants performed two squat repetitions, each lasting approximately 3 seconds. A total of three sets were completed, with three stimulation cycles per set and a 2-minute rest between sets. This protocol aims to enhance the level of neuromuscular recruitment during the conditioned activity by superimposing neuromuscular electrical stimulation onto active resistance contraction, thereby amplifying the post-activation enhancement effect observed in the SAC (Li et al., 2023).

#### 2.3.3 SSLS

Participants performed the same weighted squat protocol as in the TSLS, but electrical stimulation was applied only at the sensory threshold level, without inducing noticeable muscle contraction, to control for participants subjective expectations and placebo effects.

### 2.4 Data acquisition

This study simultaneously acquired kinematic, kinetic, and surface electromyography (EMG) signals. Because the motion capture system, force plate, and EMG system were separate devices rather than components of a single integrated system, synchronization was achieved using an external hardware trigger and subsequent time alignment. At the beginning of each valid trial, a trigger pulse was generated and sent simultaneously to the Vicon motion capture system, the Kistler force plate acquisition system, and the Delsys Trigno EMG system. This trigger signal was recorded as a common time-zero marker in each data stream. During data processing, the trigger marker was used to align the kinematic, kinetic, and EMG signals on a common time axis. The vertical ground reaction force signal was then used to identify initial contact and toe-off, and these events were applied to segment and time-normalize the corresponding kinematic and EMG data. Trials were considered valid only when the trigger marker and force-plate event timing were clearly identifiable across all systems.

#### 2.4.1 Kinematic data acquisition (motion sensor)

**Figure 1:**
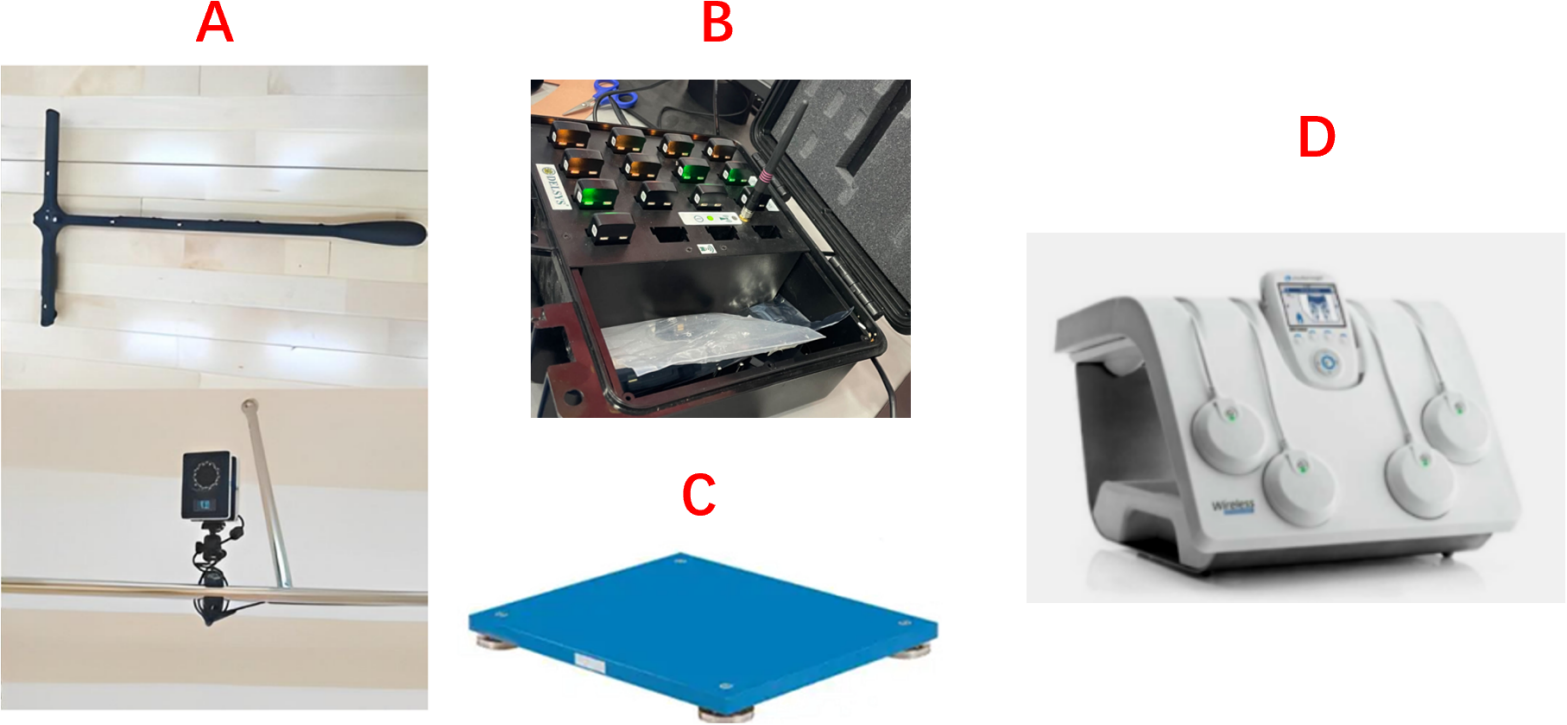
Experimental setup and workflow. **(A)** Placement of reflective markers for 3D motion capture. **(B)** Surface EMG electrode placement on target muscles. **(C)** Kistler force plate embedded in treadmill for ground reaction force measurement. **(D)** Neuromuscular electrical stimulation applied to rectus femoris and gluteus maximus during weighted squats. Arrows indicate the temporal sequence of baseline testing, group-specific intervention, 6-min recovery, and post-test. All systems were synchronized to allow simultaneous acquisition of kinematic, kinetic, and EMG data.

A 10-camera Vicon infrared 3D motion capture system (Vicon, Oxford, UK) was used to record the 3D trajectories of reflective markers at a sampling rate of 200 Hz. The placement of reflective markers followed the Plug-in-Gait and lower limb model specifications in Anybody version 7.4, covering anatomical landmarks such as the anterior and posterior points of the head, sternum, xiphoid process, T10 vertebra, anterior superior iliac spine, posterior superior iliac spine, lower third of the lateral thigh, the lateral superior condyle of the fibula, the lower third of the lateral calf, the heel, the lateral malleolus, the head of the first metatarsal, and the head of the fifth metatarsal, to obtain three-dimensional kinematic information of the pelvis and lower limb joints during the SAC. All markers were applied by the same trained researcher to ensure test consistency (**Figure 1A**).

#### 2.4.2 Surface electromyography acquisition (motion sensor)

Following skin preparation, EMG signals from the target muscle groups were recorded using the Trigno Wireless System (Delsys, Boston, USA) at a sampling rate of 2000 Hz. Prior to electrode placement, the local skin was shaved, cleaned with alcohol, and lightly abraded to reduce skin impedance. Electrode placement followed SENIAM recommendations: bipolar electrodes were positioned along the direction of muscle fibers on the muscle bellies of the target muscles, maintaining consistent electrode spacing and avoiding tendon regions as much as possible to minimize crosstalk and motion artifacts (De Luca et al., 2010). During data acquisition, electrodes were checked for loosening relative to the marked points, and any abnormal trials were recorded. Surface EMG signals were acquired synchronously with motion capture and force plate signals for subsequent analysis of RMS, iEMG, and muscle activation characteristics (**Figure 1B**).

#### 2.4.3 Kinematic data acquisition (motion sensor)

A Kistler force plate (400 × 600 mm, Type 9281, Kistler Instrument AG, Winterthur, Switzerland) was embedded in the experimental treadmill to record ground reaction force signals at a sampling rate of 1000 Hz, synchronized with the motion capture system. Gait events were extracted using the vertical ground reaction force threshold method: a vertical component exceeding 20 N was classified as initial contact (IC), while a value below 20 N was classified as toe-off (TO). Based on these events, the SAC support phase was segmented, and this segmentation was used for subsequent time alignment of joint kinematic and surface EMG signals, as well as for the extraction of analysis windows (**Figure 1C**).

#### 2.4.4 Neuromuscular electrical stimulation equipment (motion sensor)

A dual-channel neuromuscular electrical stimulator was used to deliver stimulation to the bilateral rectus femoris and gluteus maximus muscles during the TSLS intervention. The stimulation protocol was identical to that described in Section 2.3.2. The equipment was used only during the intervention and was not part of the synchronized biomechanical data acquisition system (**Figure 1D**) (Lin et al., 2025).

#### 2.4.5 Performance testing equipment

A dual-photocell timing gate was used to record V-cut test performance, with a timing accuracy of 0.01 s. This equipment was used to record the total time taken by the subject to complete the test task and served as a key external performance indicator for evaluating the post-activation enhancement effect.

### 2.5 Test tasks

#### 2.5.1 SAC test

The SAC test employs a predetermined 45° lateral cut task. Participants run 5 m in a straight line from the starting point, perform a single-leg support using their dominant lower limb, and immediately execute a 45° lateral cut in the predetermined direction, followed by continuing to accelerate for 3 m in the new direction. During the test, participants are required to keep their gaze forward and perform the movement in a smooth and natural manner, without any noticeable deceleration, hesitation, extra steps, or intentional changes to the movement pattern (Aztarain-Cardiel et al., 2024).

To minimize the impact of fluctuations in running speed on kinematic and surface electromyography (EMG) signals, a photoelectric timing gate is used to monitor the running speed during the final 2 m of the run, ensuring it remains within the participant’s preset speed range. Prior to the formal test, all subjects performed several practice runs to familiarize themselves with the testing procedure and movement requirements. During formal data collection, trials were counted as valid until each subject had obtained at least 5 valid trials.

The criteria for valid trials are as follows:

1. The subject completed the prescribed 45° lateral cut as required;
2. The dominant side’s supporting leg completes a clear and complete support and change-of-direction process;
3. The running speed is within the specified range;
4. There are no obvious pauses, dragging of the feet, or incorrect direction during the movement;
5. Signal acquisition from the motion capture system, force plate, and surface electromyography system is complete.

Invalid trials are retested on-site.

#### 2.5.2 V-cut test

The V-cut test is used to evaluate the subject’s change-of-direction performance. During the test, the subject accelerates in a straight line from the starting point, performs consecutive changes of direction along a predetermined V-shaped route, and finally sprints across the finish line. The subject is required to perform the movement with maximum effort; they must not intentionally slow down, alter the predetermined route, or exhibit noticeable pauses. A photoelectric timing system records the total test time, which serves as the V-cut test score. This test comprehensively reflects the subject’s deceleration ability, change-of-direction control, and re-acceleration ability under high-speed conditions; therefore, it is used to evaluate athletic performance related to SAC (Aztarain-Cardiel et al., 2024).

**Figure 2:**
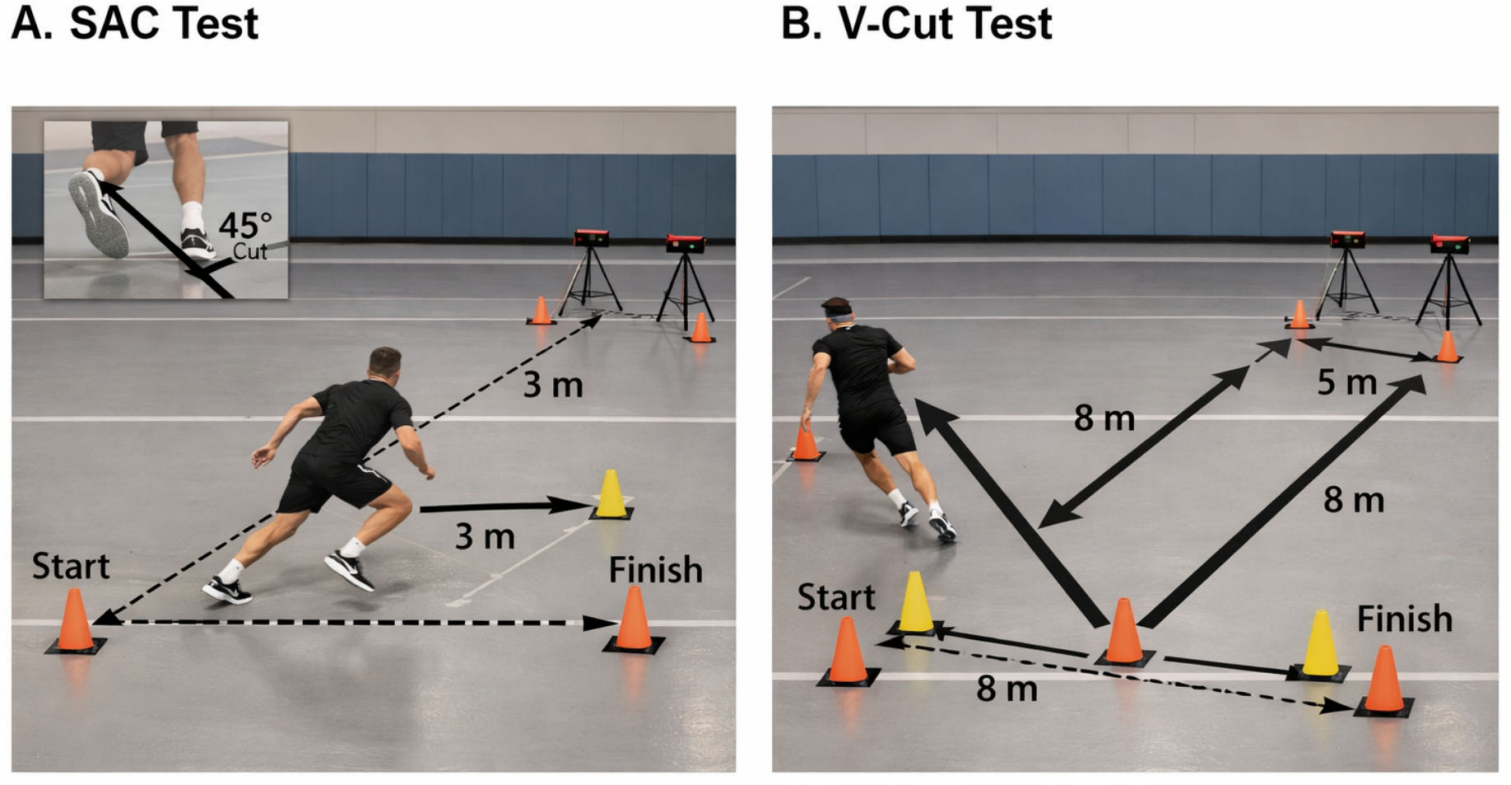
Schematic illustration of the SAC/V-cut movement task and data acquisition procedure.

Each subject completed three trials, with a 2–3-minute rest period between consecutive trials, and the best score was used for statistical analysis. To minimize the interference of fatigue and order effects, all subjects followed the same test sequence and conditions in both the pre- and post-tests (**Figure 2**).

### 2.6 Data processing

Kinematic and kinetic data were processed in Visual3D (C-Motion, USA). The three-dimensional marker trajectories were first inspected and labeled, and gaps were filled when necessary. Marker trajectory data were then filtered using a fourth-order zero-lag Butterworth low-pass filter with a cut-off frequency of 6 Hz. Ground reaction force data were filtered using a fourth-order zero-lag Butterworth low-pass filter with a cut-off frequency of 15 Hz. The use of zero-lag Butterworth filtering was intended to reduce high-frequency noise while avoiding phase shift in the time-series data. The cut-off frequencies were selected based on commonly used procedures in human movement biomechanics and previous recommendations for processing kinematic and force-plate data during dynamic movement tasks. Joint angles and gait events were then calculated in Visual3D. The processed kinematic and kinetic data were exported for subsequent calculation of lower-limb joint coordination variables in Python using custom scripts (Hermens et al., 2000).

Gait events were identified using a vertical ground reaction force threshold method: when the vertical component > 20 N, it was classified as initial contact (IC); when the vertical component< 20 N, it was classified as toe-off (TO). In this study, the complete support cycle is defined as the period from initial contact to toe-off of the dominant leg’s foot during the SAC. This cycle is time-normalized (0%–100%) to enable temporal alignment and subsequent dynamic comparisons across trials and participants.

Given that the SAC support phase is brief and contains critical information regarding deceleration control, postural adjustment, and preparation for re-acceleration shortly after ground contact, this study further integrated the results from the full support cycle to identify key phases of the support phase for subsequent analysis of lower limb joint coordination and surface electromyography. To further describe the phasic changes in neuromuscular control characteristics within the SAC support phase, the full support cycle was divided into three phases: the initial braking phase, the cushioning control phase, and the propulsion-takeoff phase:

1. Initial braking phase: From initial contact to the early support phase, primarily reflecting the initial deceleration process following contact with the ground, To objectively define the subphases of the SAC support phase, the full support phase was first defined as the period from initial contact (IC) to toe-off (TO) of the dominant leg and was time-normalized to 0%–100%. The cushioning control phase was centered around the instant of maximum knee flexion, which was identified from the knee flexion angle time series. Accordingly, the initial braking phase was defined as the period from IC to maximum knee flexion, and the propulsion–takeoff phase was defined as the period from maximum knee flexion to TO. This event-based definition avoided the ambiguous use of the term “early support phase” and provided an objective criterion for phase segmentation (Jòdar-Portas et al., 2023);
2. Cushioning control phase: From the early support phase to the moment of maximum knee flexion, primarily reflecting the lower limb’s control of the body’s center of gravity descent and postural stability;
3. Propulsion and takeoff phase: From the moment of maximum knee flexion to takeoff, primarily reflecting the propulsion process of the body re-accelerating in a new direction (Li et al., 2025).

For the SAC task, each participant performed at least five valid trials, and the mean value was calculated for analysis. This approach was adopted because SAC is a complex multi-joint movement involving rapid braking, stabilization, and re-acceleration, and single-trial performance may be influenced by trial-to-trial variability. Using the mean of multiple trials provides a more reliable estimate of typical task performance. For the V-cut test, participants performed three trials, and the best performance was used for analysis. The V-cut test is a simpler, short-duration change-of-direction sprint, and using the best trial reflects the participant’s maximal capability while minimizing the influence of fatigue across repeated trials. This rationale justifies the different trial reduction strategies for the two tasks.

### 2.7 Data metrics

#### 2.7.1 Calculation of lower limb joint coordination

Inter-joint coordination analysis was used to describe how adjacent lower-limb joints worked together during the SAC support phase. Unlike single-joint kinematic variables, which describe the movement of one joint independently, coordination analysis reflects the relationship between two joints over time. This is important for the SAC task because braking, body stabilization, and re-acceleration require the hip, knee, and ankle joints to act in a coordinated manner rather than as isolated segments. Therefore, inter-joint coordination provides information about the movement control strategy used by the athlete during direction change.

In this study, vector coding was used to quantify the coordination patterns of the hip–knee, knee–ankle, and hip–ankle joint couplings. Briefly, angle–angle plots were constructed from sagittal-plane joint angle time series, and the direction of change between consecutive data points was used to calculate a coupling angle. The coupling angle indicates which joint contributes more to the movement at a given moment and whether the two joints move in a similar or opposite direction. In-phase coordination indicates that two joints move in a similar direction, anti-phase coordination indicates that they move in opposite directions, proximal-phase coordination indicates greater contribution from the proximal joint, and distal-phase coordination indicates greater contribution from the distal joint. In this way, vector coding allows the dynamic coordination strategy during SAC to be described in an interpretable and task-specific manner.

To calculate coordination between lower limb joints, this study employed a modified vector coding method to compute joint coupling angles and classify coordination patterns. Compared to the continuous phase method, the vector coding method is characterized by its simplicity of implementation, strong applicability to various movement tasks, and ease in describing coordination relationships between adjacent joints (Cimolato et al., 2022).

First, sagittal-plane hip, knee, and ankle joint angles were exported from Visual3D for vector coding analysis. Specifically, hip flexion–extension, knee flexion–extension, and ankle dorsiflexion–plantarflexion angle time series were used to construct angle–angle plots for the hip–knee, knee–ankle, and hip–ankle joint couplings. Vector coding was performed as a single-plane analysis using only sagittal-plane joint angle time series. No cross-plane or three-dimensional vector coding analysis was performed in this study. The sagittal plane was selected because the SAC support phase involves rapid braking, cushioning, and propulsion, which are primarily reflected by flexion–extension control of the lower-limb joints. All angle time series were segmented based on the SAC full support cycle and time-normalized to ensure comparability across trials on the same relative time scale. Subsequently, “angle-angle plots” were constructed to describe the cooperative changes between two joints within the same cycle. For any two angle sequences θ1(t) and θ2(t), a vector was constructed between adjacent time points ti and ti+1:

Δθ1=θ1(ti+1)−θ1(ti).


Δθ2=θ2(ti+1)−θ2(ti)

The coupling angle is defined as the angle between this vector and the horizontal axis:


ϕ(ti)=atan2(Δθ2,Δθ1)

We uniformly map ϕ(ti) to the range of 0°–360°. In this way, ϕ(ti) at each time point represents the coordination state of the two joints at that moment; by concatenating all ϕ(t) values across the entire cycle, we obtain the coordination time series of the joint coupling over the entire support cycle.

This study focuses on analyzing the coordination characteristics of joint couplings such as hip-knee and knee-ankle, and classifies coordination patterns into four categories based on the range of values for the coupling angle ϕ(t):

1. In-phase coordination: ϕ ∈ [22.5°, 67.5°] or [202.5°, 247.5°], indicating that the two joints rotate synchronously in the same direction;
2. Anti-phase coordination: ϕ ∈ [112.5°, 157.5°] or [292.5°, 337.5°], indicating that the two joints rotate primarily in opposite directions;
3. Proximal phase: ϕ ∈ [157.5°, 202.5°] or [337.5°, 360°], indicating that the movement is primarily contributed by the proximal joint;
4. Distal phase: ϕ ∈ [67.5°, 112.5°] or [247.5°, 292.5°], indicating that movement is primarily contributed by the distal joint.

In the quantitative analysis, this study extracted the average coupling angles, coordination variability, and the proportion of different coordination patterns for each joint during the entire stance phase and its key stages. These metrics were used to compare changes in lower limb joint coordination control characteristics within the SAC under different intervention conditions.

#### 2.7.2 Surface electromyography processing

Before the formal SAC test, maximal voluntary contraction (MVC) tests were performed for each recorded muscle to obtain an individual reference EMG amplitude for normalization. For each target muscle, participants completed three MVC trials under standardized testing positions, with each contraction lasting approximately 5 s and with sufficient rest between trials to minimize fatigue. During each trial, participants were instructed to gradually increase force and then maintain maximal effort. The stable portion of each MVC trial was selected for analysis, and the highest EMG amplitude obtained across the three trials was used as the MVC reference value for that muscle (Hermens et al., 2000).

After band-pass filtering and full-wave rectification, the EMG signals recorded during the SAC task were normalized using the corresponding MVC reference value for each muscle and participant. This MVC-based normalization was applied before calculating RMS and iEMG, so that the extracted EMG metrics reflected activation levels adjusted for individual differences in signal amplitude. The purpose of this procedure was to reduce inter-individual variability caused by differences in skin impedance, electrode placement, subcutaneous tissue thickness, and signal gain, while preserving the original metric definitions and unit presentation for RMS and iEMG.

Raw surface EMG signals were processed separately from the kinematic and kinetic data using custom Python scripts. The EMG signals were first band-pass filtered between 20 and 400 Hz using a fourth-order zero-phase Butterworth filter to reduce low-frequency motion artifacts and high-frequency noise while retaining the main frequency components of the surface EMG signal. The filtered EMG signals were then full-wave rectified. This processing procedure was selected according to commonly used surface EMG processing recommendations and previous studies indicating that a band-pass range beginning at approximately 20 Hz is appropriate for reducing motion artifacts in dynamic tasks. The rectified EMG signals were segmented according to the SAC support phase and subphases defined in Section 2.6. After segmentation, the EMG data were time-normalized using linear interpolation to allow comparisons across trials and participants. RMS and iEMG were then calculated for each target muscle during the full support phase and predefined subphases (Avila et al., 2023).

This study extracted the root mean square (RMS) and integrated electromyography (iEMG) metrics for the target muscle groups during the support phase. RMS was used to reflect changes in EMG signal amplitude and the instantaneous activation intensity of muscle groups within a specific time window, while iEMG was used to reflect the overall level of electrical activity of muscle groups over a certain time range. RMS and iEMG were calculated for each target muscle during the full SAC support phase and the predefined subphases described in Section 2.6.

Let the electromyographic signal of a target muscle group within an analysis time window of length N be denoted as xi (i = 1, 2, …, N). Then, the formula for calculating the RMS is:


$$RMS=\sqrt{\frac{1}{N}\sum_{i=1}^{N}x_{i}^{2}}$$

Here, xi represents the amplitude of the EMG signal at the i-th sampling point, and N represents the total number of sampling points within the analysis window. A higher RMS value indicates a higher overall amplitude of the EMG signal within that time window and a stronger instantaneous activation level of the muscle group. The iEMG is calculated based on the rectified EMG signal, and its formula is:


$$iEMG=\int_{t_{1}}^{t_{2}}|EMG\left(t\right)|dt$$

In discrete form, this can be expressed as:


$$iEMG=\sum_{i=1}^{N}|x_{i}|\text{Δ}t$$

Here, |xi| represents the amplitude of the rectified EMG signal at the i-th sampling point, and Δt denotes the time interval between adjacent sampling points. A higher iEMG value indicates a higher overall level of electrical activity in the muscle group during that analysis phase.

The target muscle groups recorded in this study were selected based on their functional roles in SAC performance. Lower-limb muscles, including the gluteus maximus (GM), vastus medialis (VM), biceps femoris (BF), tibialis anterior (TA), and gluteus medius (GLM), are directly involved in braking, stabilization, and re-acceleration. Core muscles, including the rectus abdominis (ABS) and latissimus dorsi (LD), support trunk stabilization and inter-segmental force transfer. Upper-limb muscles, such as biceps brachii (BB), brachioradialis (BRD), and pectoralis major (PM), contribute to arm swing and balance during directional changes. Recording this comprehensive set of muscles allows for the assessment of how the combined NMES and weighted squat intervention acutely affects both primary and auxiliary muscles involved in SAC, providing a holistic view of neuromuscular coordination and task-specific activation patterns (Hermens et al., 2000).

Considering the differences in the functional roles of various muscle groups during the SAC, this study further combined the phases of the stance cycle to conduct a comparative analysis of the RMS and iEMG of each muscle group across different phases, in order to reveal the recruitment patterns of relevant upper and lower limb and trunk muscle groups in movement control following the combined intervention of neuromuscular electrical stimulation and weighted squats (Li et al., 2025).

### 2.8 Statistical analysis

Statistical analysis was performed using SPSS 26.0 software (SPSS Inc., Chicago, IL, USA), with a significance level set at α = 0.05. For each participant, the mean of the valid trials was calculated before proceeding to group-level statistical analysis. All results are expressed as mean ± standard deviation (x̄ ± s).

Before inferential statistical analysis, the normality of each dependent variable was assessed using the Shapiro–Wilk test, and the homogeneity of variance among groups was assessed using Levene’s test. For the mixed-design ANOVA, the within-subject factor was time, with only two levels (pre-test and post-test). Therefore, the assumption of sphericity was automatically satisfied and Mauchly’s test of sphericity was not required. If a repeated-measures factor with more than two levels had been analyzed, Mauchly’s test would have been used and Greenhouse–Geisser correction would have been applied when the sphericity assumption was violated.

For V-cut test scores, a 3-group × 2-time (pre/post) mixed-design analysis of variance was employed to test for main effects of group and time, as well as the group × time interaction, with F-values, P-values, and effect sizes (η²) reported (Hambly et al., 2023).

For the surface electromyography (EMG) metrics extracted from the SAC test [i.e., root mean square (RMS) and integrated EMG (iEMG) of each target muscle group during the full support phase and its critical stages] and lower limb joint coordination metrics [i.e., average coupling angle, coordination variability, and the proportion of different coordination patterns for each joint during the full support phase and its critical stages], a 3-group × 2-time (pre/post) mixed-design ANOVA was also employed to test for main effects of group and time, as well as the group × time interaction, with F-values, P-values, and effect sizes (η²) reported.

*Post hoc* tests were conducted only when the group × time interaction effect reached statistical significance (P< 0.05). This approach follows standard procedures for mixed-design ANOVA, where *post hoc* comparisons are justified only when the interaction indicates that the pattern of change over time differs between groups. Conducting *post hoc* tests without a significant interaction may lead to misleading conclusions, as the main effects alone do not capture group-specific temporal changes. Within-group pre–post comparisons were performed using paired t-tests, reporting t-values, P-values, and Cohen’s d; between-group comparisons at each time point were conducted using one-way ANOVA with Bonferroni correction for multiple comparisons as appropriate. All statistical tests were two-tailed.

## 3 Results

### 3.1 Comparison of V-cut test performance results before and after the intervention

There were no statistically significant differences in V-cut test scores among the three groups prior to the intervention. After the intervention, only the TSLS group showed a significant improvement compared with its pre-intervention value and was significantly lower than both the LS and SSLS groups. No significant pre–post changes were observed in the LS or SSLS groups. The V-cut test showed a significant effect (F = 16.27, P< 0.001, η² = 0.606) (**Table 2**).

**Table 2:** V-cut test performance before and after the intervention (M ± SD).

Indicator	Group						F value	P value	η²
V-cut test	LS		SSLS		TSLS		16.27	<0.001	0.606
	pre	post	pre	post	pre	post			
	4.86 ± 0.14	4.83 ± 0.13	4.87 ± 0.15	4.82 ± 0.14	4.85 ± 0.13	4.68 ± 0.12^a,b,c^			

LS, loaded squat group; SSLS, sham stimulation combined with loaded squat group; TSLS, true stimulation combined with loaded squat group.

^a^ indicates a significant difference compared with pre within the same group (P< 0.05).

^b^ indicates a significant difference compared with post in the LS group (P< 0.05).

^c^ indicates a significant difference compared with post in the SSLS group (P< 0.05).

Lower V-cut test values indicate better change-of-direction performance.

### 3.2 Results of muscle activation during the SAC task

#### 3.2.1 Comparison of RMS results during the full support phase

There were no statistically significant differences in the RMS values of the target muscle groups across all three groups during the full support phase prior to the intervention. Results from a mixed-design analysis of variance indicated a significant group × time interaction for the RMS values of the GM, VM, and BF, specifically for the GM (F = 14.62, P<0.001, η²=0.587), VM (F = 13.48, P<0.001, η²=0.565), and BF (F = 12.91, P<0.001, η²=0.551). After the intervention, the RMS values for TSLS, GM, VM, and BF were 10.8 ± 1.8, 10.4 ± 1.7, and 9.8 ± 1.6, respectively, all of which were significantly higher than the pre-intervention values in this group and also significantly higher than the corresponding post-intervention values for LS and SSLS (P< 0.05). LS and SSLS also showed a certain upward trend in the muscle groups but did not reach the same level.

For the BB, BRD, TA, ABS, LD, GLM, and PM, the group × time interaction did not reach statistical significance (P > 0.05). Although the RMS values of all muscle groups were generally higher after the TSLS intervention than before, the magnitude of change was limited and did not show significant statistical differences. The results suggest that significant changes in RMS during the full support phase were primarily concentrated in the GM, VM, and BF (**Table 3**).

**Table 3:** Comparison of RMS during the full support period before and after the intervention (M ± SD, unit: μV).

Muscle	Group						F-value	P-value	η²
	LS		SSLS		TSLS				
	Pre	Post	Pre	Post	Pre	Post			
BB	6.1 ± 1.5	6.3 ± 1.5	6.4 ± 1.6	6.7 ± 1.6	6.6 ± 1.5	7.0 ± 1.5	1.42	0.256	0.079
BRD	5.3 ± 1.4	5.5 ± 1.4	5.6 ± 1.5	5.9 ± 1.5	5.8 ± 1.4	6.2 ± 1.4	1.37	0.271	0.076
TA	5.0 ± 1.4	5.2 ± 1.4	5.2 ± 1.5	5.5 ± 1.5	5.4 ± 1.4	5.8 ± 1.4	1.65	0.208	0.091
GM	7.8 ± 1.8	8.0 ± 1.8	8.4 ± 1.9	9.2 ± 1.9	8.3 ± 1.8	10.8 ± 1.8abc	14.62	<0.001	0.587
VM	7.2 ± 1.7	7.5 ± 1.7	7.8 ± 1.8	8.8 ± 1.8	7.9 ± 1.7	10.4 ± 1.7abc	13.48	<0.001	0.565
BF	6.6 ± 1.6	6.9 ± 1.6	7.1 ± 1.7	8.2 ± 1.7	7.2 ± 1.6	9.8 ± 1.6abc	12.91	<0.001	0.551
ABS	6.3 ± 1.5	6.5 ± 1.5	6.5 ± 1.6	6.8 ± 1.6	6.8 ± 1.5	7.1 ± 1.5	1.28	0.291	0.071
LD	8.0 ± 1.9	8.2 ± 1.9	8.2 ± 2.0	8.5 ± 2.0	8.4 ± 1.9	8.8 ± 1.9	1.11	0.342	0.063
GLM	7.6 ± 1.8	7.8 ± 1.8	7.8 ± 1.9	8.1 ± 1.9	8.0 ± 1.8	8.4 ± 1.8	1.24	0.301	0.069
PM	6.0 ± 1.5	6.2 ± 1.5	6.2 ± 1.6	6.5 ± 1.6	6.4 ± 1.5	6.8 ± 1.5	1.33	0.283	0.074

LS, loaded squat group; SSLS, sham stimulation combined with loaded squat group; TSLS, true stimulation combined with loaded squat group. a indicates a significant difference compared to the pre-test within this group (P< 0.05); b indicates a significant difference compared to the post-test in the LS group (P< 0.05); c indicates a significant difference compared to SSLS post (P< 0.05). F-values, P-values, and η² represent the results of the group × time interaction.

#### 3.2.2 Comparison of iEMG results during the full support phase

The iEMG values for the target muscle groups during the full support phase prior to intervention were generally similar across the three groups, with no statistically significant differences between groups. Results from a mixed-design analysis of variance (ANOVA) revealed significant group × time interactions for the GM, VM, and BF before and after the intervention, specifically for the GM (F = 15.26, P< 0.001, η² = 0.601), VM (F = 14.11, P<0.001, η²=0.578), and BF (F = 13.37, P<0.001, η²=0.562). Specifically, following the TSLS intervention, the iEMG values for GM, VM, and BF increased to 10.2 ± 2.0, 9.8 ± 1.9, and 9.3 ± 1.8, respectively, all of which were significantly higher than pre-intervention levels in this group and post-intervention levels in the LS and SSLS groups (P< 0.05). In contrast, although LS and SSLS showed a certain upward trend in the muscle groups, the magnitude of change was relatively small.

The iEMG values of the remaining muscle groups, including BB, BRD, TA, ABS, LD, GLM, and PM, also showed a slight upward trend before and after the intervention, but the group × time interaction did not reach statistical significance (F = 1.17–1.58, P > 0.05, η² = 0.066–0.087). Specifically, for BB, the values after LS, SSLS, and TSLS interventions were 5.2 ± 1.8, 5.5 ± 1.9, and 5.8 ± 1.7, respectively; for TA, the values after intervention were 4.5 ± 1.6, 4.8 ± 1.7, and 5.1 ± 1.5, respectively; and 7.5 ± 2.2, 7.8 ± 2.3, and 8.1 ± 2.1, respectively, following LD intervention; none of these showed significant differences between groups.

Overall, the iEMG results from the full support period indicate that significant changes were primarily concentrated in the three key muscle groups—GM, VM, and BF—while the remaining muscle groups showed some degree of increase before and after the intervention, but these changes did not reach statistical significance (**Table 4**).

**Table 4:** Comparison of iEMG during the full support period before and after the intervention (M ± SD, unit: μV·s).

Muscle	Group						F-value	P-value	η²
	LS		SSLS		TSLS				
	Pre	Post	Pre	Post	Pre	Post			
BB	5.0 ± 1.8	5.2 ± 1.8	5.3 ± 1.9	5.5 ± 1.9	5.5 ± 1.7	5.8 ± 1.7	1.36	0.273	0.076
BRD	4.7 ± 1.7	4.9 ± 1.7	4.9 ± 1.8	5.2 ± 1.8	5.1 ± 1.6	5.5 ± 1.6	1.41	0.258	0.078
TA	4.3 ± 1.6	4.5 ± 1.6	4.5 ± 1.7	4.8 ± 1.7	4.7 ± 1.5	5.1 ± 1.5	1.58	0.221	0.087
GM	6.9 ± 2.1	7.2 ± 2.1	7.3 ± 2.2	8.5 ± 2.2	7.4 ± 2.0	10.2 ± 2.0abc	15.26	<0.001	0.601
VM	6.4 ± 2.0	6.8 ± 2.0	6.9 ± 2.1	8.1 ± 2.1	7.0 ± 1.9	9.8 ± 1.9abc	14.11	<0.001	0.578
BF	5.9 ± 1.9	6.2 ± 1.9	6.3 ± 2.0	7.6 ± 2.0	6.5 ± 1.8	9.3 ± 1.8abc	13.37	<0.001	0.562
ABS	5.5 ± 1.8	5.8 ± 1.8	5.8 ± 1.9	6.1 ± 1.9	6.1 ± 1.7	6.4 ± 1.7	1.29	0.288	0.072
LD	7.2 ± 2.2	7.5 ± 2.2	7.5 ± 2.3	7.8 ± 2.3	7.8 ± 2.1	8.1 ± 2.1	1.17	0.324	0.066
GLM	6.7 ± 2.0	7.0 ± 2.0	7.0 ± 2.1	7.3 ± 2.1	7.3 ± 1.9	7.6 ± 1.9	1.22	0.307	0.068
PM	5.2 ± 1.7	5.5 ± 1.7	5.5 ± 1.8	5.8 ± 1.8	5.8 ± 1.6	6.1 ± 1.6	1.31	0.284	0.073

LS, loaded squat group; SSLS, sham stimulation combined with loaded squat group; TSLS, true stimulation combined with loaded squat group. a indicates a significant difference compared to the pre-test within this group (P< 0.05); b indicates a significant difference compared to the post-test in the LS group (P< 0.05); c indicates a significant difference compared to SSLS post (P< 0.05). F-values, P-values, and η² represent the results of the group × time interaction.

### 3.3 Comparison of lower limb joint coordination results in the SAC task

#### 3.3.1 Comparison of hip-knee joint coordination results

In the hip-knee coordination patterns, the main effect of group for in-phase coordination was not significant (P = 0.456, η² = 0.07), while the main effect of time was significant (P = 0.012, η² = 0.26), and the group × time interaction was significant (P< 0.001, η² = 0.58). In terms of within-group trends, the proportion of in-phase coordination decreased in both the LS and SSLS groups after the intervention, while the TSLS group showed an upward trend (See **Table 5** for results; see **Supplementary Appendix 1** for the analysis of main effects).

**Table 5:** Results of the analysis of variance for the proportion of hip-knee coordination patterns (M ± SD, unit: %).

Coordination mode	Group	Pre	Post	Main effect of group	Time main effect	Group × time interaction
In phase	LS	9.809 ± 1.912	6.851 ± 0.592	P=0.456, η²=0.07	P=0.012*, η²=0.26	P<0.001*, η²=0.58
	SSLS	12.486 ± 4.331	6.715 ± 0.809			
	TSLS	7.892 ± 3.623	9.977 ± 3.781			
reverse phase	LS	51.046 ± 19.430	66.597 ± 6.241	P=0.984, η²=0.00	P=0.027*, η²=0.21	P=0.506, η²=0.06
	SSLS	59.590 ± 7.988	65.394 ± 3.814			
	TSLS	57.802 ± 12.095	63.618 ± 6.691			
proximal	LS	24.949 ± 26.684	12.968 ± 4.739	P=0.231, η²=0.13	P=0.032*, η²=0.20	P=0.285, η²=0.11
	SSLS	17.144 ± 6.855	11.870 ± 2.304			
	TSLS	14.911 ± 7.521	16.471 ± 8.999			
distal	LS	14.195 ± 7.597	13.584 ± 4.090	P=0.013*, η²=0.34	P=0.001*, η²=0.40	P<0.001*, η²=0.86
	SSLS	10.781 ± 3.282	16.020 ± 4.162			
	TSLS	19.395 ± 6.838	9.934 ± 2.533			

*LS, loaded squat group; SSLS, sham stimulation combined with loaded squat group; TSLS, true stimulation combined with loaded squat group. Indicates a statistically significant difference (P< 0.05).

For the out-of-phase coordination pattern, only the main effect of time was significant (P = 0.027, η² = 0.21); neither the main effect of group nor the group × time interaction was significant (P > 0.05). The proportion of counter-phase coordination in all three groups showed an upward trend after the intervention.

For the proximal-phase coordination pattern, only the main effect of time was significant (P = 0.032, η² = 0.20); neither the main effect of group nor the group × time interaction was significant (P > 0.05). Specifically, the proportion of proximal-phase coordination decreased in LS and SSLS after the intervention, while it increased slightly in TSLS.

For the distal coordination pattern, the main effect of group (P = 0.013, η² = 0.34), the main effect of time (P = 0.001, η² = 0.40), and the group × time interaction (P< 0.001, η² = 0.86) were all significant. Specifically, LS showed little change before and after the intervention, SSLS exhibited an upward trend after the intervention, while TSLS showed a significant decline after the intervention.

#### 3.3.2 Comparison of hip-ankle coordination results

In the hip-ankle coordination pattern, only the main effect of time was significant for in-phase coordination (P = 0.027, η² = 0.21); neither the main effect of group nor the group × time interaction was significant (P > 0.05). In terms of trends within groups, the proportion of in-phase coordination decreased after the intervention in both the LS and TSLS groups, while it increased slightly in the SSLS group.

For the counter-phase coordination pattern, neither the main effect of group, the main effect of time, nor the group × time interaction was significant (P > 0.05). Specifically, the proportion of counter-phase coordination slightly decreased in the LS group after the intervention, while it slightly increased in the SSLS and TSLS groups.

For the proximal synchronous coordination pattern, only the main effect of time was significant (P = 0.028, η² = 0.21); the main effect of group and the group × time interaction were both insignificant (P > 0.05). Specifically, the proportion of proximal synchronous coordination decreased in LS and SSLS after the intervention, while it increased slightly in TSLS.

For distal synchrony patterns, the main effect of time was significant (P = 0.011, η²=0.27), the group × time interaction was significant (P = 0.006, η²=0.39), and the main effect of group was not significant (P = 0.085, η²=0.21). In terms of trends, the proportion of distal coordination increased for both LS and TSLS after the intervention, while the change for SSLS was relatively small. (See **Table 6** for results; see **Supplementary Appendix 1** for the analysis of main effects).

**Table 6:** Results of the analysis of variance for the proportions of hip-ankle coordination patterns (M ± SD, unit: %).

Coordination mode	Group	Pre	Post	Main effect of group	Time main effect	Group × time interaction
In phase	LS	42.549 ± 13.708	31.167 ± 9.309	P=0.314, η²=0.10	P=0.027*, η²=0.21	P=0.151, η²=0.16
	SSLS	27.659 ± 12.479	31.807 ± 11.534			
	TSLS	38.163 ± 9.353	28.897 ± 7.102			
reverse phase	LS	9.596 ± 2.748	8.760 ± 2.216	P=0.637, η²=0.04	P=0.116, η²=0.11	P=0.155, η²=0.16
	SSLS	7.148 ± 7.574	9.948 ± 6.086			
	TSLS	8.526 ± 3.702	10.517 ± 4.792			
proximal	LS	10.895 ± 2.064	6.265 ± 3.248	P=0.091, η²=0.20	P=0.028*, η²=0.21	P=0.162, η²=0.16
	SSLS	7.869 ± 6.405	4.567 ± 3.655			
	TSLS	6.904 ± 2.816	7.806 ± 3.965			
distal	LS	36.959 ± 10.811	53.808 ± 9.561	P=0.085, η²=0.21	P=0.011*, η²=0.27	P=0.006*, η²=0.39
	SSLS	57.324 ± 19.435	53.679 ± 17.100			
	TSLS	46.406 ± 8.833	52.780 ± 7.526			

*LS, loaded squat group; SSLS, sham stimulation combined with loaded squat group; TSLS, true stimulation combined with loaded squat group. Indicates a statistically significant difference (P< 0.05).

#### 3.3.3 Comparison of knee-ankle coordination results

In the knee-ankle coordination patterns, the main effect of group for in-phase coordination was significant (P = 0.002, η² = 0.44), the group × time interaction was significant (P< 0.001, η² = 0.58), and the main effect of time was not significant (P = 0.216, η² = 0.07). In terms of within-group trends, the proportion of in-phase coordination in the LS and SSLS groups showed little change or a slight decrease after the intervention, while that in the TSLS group increased significantly after the intervention.

The main effects of group (P = 0.043, η²=0.26) and time (P = 0.002, η²=0.36) on the counter-phase coordination pattern, as well as the group × time interaction (P = 0.002, η²=0.44), were all significant. Specifically, the proportion of counter-phase coordination decreased for LS and TSLS after the intervention, while SSLS showed an upward trend.

For the proximal coordination pattern, the main effect of group was significant (P = 0.002, η²=0.44), the group × time interaction was significant (P<0.001, η²=0.59), and the main effect of time was not significant (P = 0.142, η²=0.10). Specifically, the proportion of proximal coordination decreased for LS and TSLS after the intervention, while it increased for SSLS.

For the distal coordination pattern, the main effect of time was significant (P< 0.001, η² = 0.57), the group × time interaction was significant (P = 0.003, η² = 0.43), and the main effect of group was not significant (P = 0.490, η² = 0.07). In terms of within-group trends, the proportion of distal phase coordination increased for LS and TSLS after the intervention, while it decreased slightly for SSLS. (See **Table 7** for results; see **Supplementary Appendix 1** for the analysis of main effects).

**Table 7:** Results of the analysis of variance for the proportions of knee-ankle coordination patterns (M ± SD, unit: %).

Coordination mode	Group	Pre	Post	Main effect of group	Time main effect	Group × time interaction
In phase	LS	7.136 ± 4.014	5.434 ± 2.350	P=0.002*, η²=0.44	P=0.216, η²=0.07	P<0.001*, η²=0.58
	SSLS	3.114 ± 0.873	2.864 ± 1.723			
	TSLS	3.894 ± 2.842	9.142 ± 5.830			
reverse phase	LS	38.787 ± 11.464	28.888 ± 10.393	P=0.043*, η²=0.26	P=0.002*, η²=0.36	P=0.002*, η²=0.44
	SSLS	30.123 ± 16.001	34.190 ± 9.364			
	TSLS	39.978 ± 13.461	24.439 ± 17.209			
proximal	LS	7.086 ± 1.373	5.367 ± 3.579	P=0.002*, η²=0.44	P=0.142, η²=0.10	P<0.001*, η²=0.59
	SSLS	5.977 ± 1.577	7.898 ± 3.722			
	TSLS	6.551 ± 4.079	3.330 ± 3.508			
distal	LS	46.991 ± 11.086	60.311 ± 10.872	P=0.490, η²=0.07	P<0.001*, η²=0.57	P=0.003*, η²=0.43
	SSLS	60.786 ± 15.852	55.048 ± 12.203			
	TSLS	49.577 ± 13.007	63.089 ± 17.248			

*LS, loaded squat group; SSLS, sham stimulation combined with loaded squat group; TSLS, true stimulation combined with loaded squat group. Indicates a statistically significant difference (P< 0.05).

## 4 Discussion

### 4.1 Effects of NMES combined with weighted squats on direct SAC-related performance

The present study found that only the TSLS group showed a significant improvement in V-cut test performance after the intervention, whereas no significant pre–post changes were observed in the LS or SSLS groups. This result suggests that the acute improvement in change-of-direction performance was more evident when NMES was combined with weighted squats than when weighted squats were performed alone or with sham stimulation. However, this finding should not be interpreted simply as evidence that the combined intervention directly “improved” all aspects of SAC movement capacity. Rather, it indicates that, under the present experimental conditions, the combined mechanical and electrical stimulus was associated with a short-term performance advantage in a basketball-relevant directional-change task (Aztarain-Cardiel et al., 2025).

From a mechanistic perspective, SAC-related performance depends on the ability to rapidly decelerate, stabilize the body, redirect the center of mass, and re-accelerate within a short support phase (Baena-Raya et al., 2024). Weighted squats may provide a mechanical conditioning stimulus to the hip and knee extensor system, while NMES may additionally increase peripheral neuromuscular excitation in the stimulated muscles. Therefore, the performance improvement observed in the TSLS group may reflect a more favorable acute balance between potentiation and fatigue. In this context, the combined intervention may have increased neuromuscular readiness before the post-test, allowing athletes to produce and coordinate force more effectively during the subsequent change-of-direction task.

Nevertheless, the present findings cannot identify a single physiological mechanism responsible for the improved V-cut performance. The improvement may be partly related to increased lower-limb muscle activation, altered inter-joint coordination, greater arousal, or a short-term change in movement strategy. Although the use of sham stimulation helped control for expectation-related effects to some extent, expectancy and acute arousal cannot be completely excluded. Therefore, the performance result should be interpreted as an acute task-level response to the combined intervention rather than direct proof of a specific neural or muscular mechanism.

Compared with commonly used PAPE tasks such as vertical jumps, sprint drills, or general resistance exercises, weighted squats combined with NMES may be more relevant to SAC preparation because they provide both a multi-joint mechanical stimulus and an additional electrical stimulus to muscles involved in lower-limb support and propulsion. Weighted squats emphasize hip and knee extension, which is related to force absorption and force re-generation during directional change. NMES may further increase excitation in selected target muscles during the conditioning task. However, this does not mean that the combined intervention is universally superior to other PAPE strategies. Its advantage may depend on the characteristics of the subsequent task, the athlete’s training background, the stimulation parameters, and the recovery interval. Thus, the present study supports the potential task-specific relevance of NMES combined with weighted squats for SAC-related performance, but further comparisons with other conditioning activities are still needed.

### 4.2 Effects of NMES combined with weighted squats on muscle activation during the SAC task

The present study found that significant changes in RMS and iEMG were mainly concentrated in the GM, VM, and BF in the TSLS group. These muscles are functionally relevant to braking, support, stabilization, and re-acceleration during SAC. Therefore, the observed increases in EMG amplitude-based variables may indicate that the combined intervention acutely changed the activation level of key muscles involved in the task (Jimenez-Iglesias et al., 2024).

However, the interpretation of RMS and iEMG requires caution. RMS reflects the amplitude-related characteristics of the EMG signal within a given time window, while iEMG reflects the accumulated electrical activity over the analyzed period. Higher RMS or iEMG values may be associated with greater motor unit recruitment, increased firing rate, or greater neural drive, but these variables cannot distinguish among these mechanisms directly. Therefore, the present results should be interpreted as evidence of altered muscle activation patterns rather than definitive evidence of improved motor unit recruitment efficiency.

The fact that the significant EMG changes were concentrated in GM, VM, and BF may have task-specific relevance. During the SAC support phase, the GM contributes to hip extension and pelvic control, the VM contributes to knee stabilization and force transmission, and the BF contributes to posterior-chain control during braking and redirection. The selective increase in these muscles may suggest that NMES combined with weighted squats influenced muscles that are more directly involved in the mechanical demands of SAC. This supports the idea that the combined intervention did not simply produce a generalized increase in EMG activity across all recorded muscles but rather produced a more localized response in muscles closely related to the task (Fort-Vanmeerhaeghe et al., 2022).

At the same time, greater EMG amplitude should not automatically be interpreted as better movement efficiency. Increased RMS or iEMG may reflect a beneficial increase in neuromuscular readiness, but it may also reflect higher neuromuscular effort, compensatory recruitment, or fatigue-related changes. Because this study did not include frequency-domain EMG variables, such as median frequency or mean frequency, it was not possible to determine whether the EMG amplitude changes were accompanied by fatigue-related spectral shifts (Madruga-Parera et al., 2022). Therefore, the present EMG findings provide useful information about activation amplitude, but they do not allow direct conclusions about fatigue status, motor unit conduction velocity, or firing behavior.

This point is important because NMES can theoretically increase both potentiation and fatigue. The present intervention included a moderate load, low repetition volume, rest intervals between sets, and a 6-min recovery period to reduce excessive fatigue. However, fatigue was not directly quantified. Therefore, while the improved V-cut performance and the selective increase in EMG amplitude suggest that excessive fatigue was unlikely to dominate the post-test response, the possibility of subclinical or muscle-specific fatigue cannot be excluded. Future studies should include EMG frequency-domain analysis, force decrement measures, perceived exertion, or metabolic indicators to better separate potentiation-related activation from fatigue-related responses (Marín et al., 2026).

### 4.3 Effects of NMES combined with weighted squats on lower limb joint coordination during the SAC task

The present study also found that NMES combined with weighted squats influenced sagittal-plane lower-limb joint coordination during the SAC task, particularly in the hip–knee and knee–ankle couplings. These findings suggest that the combined intervention was associated not only with changes in muscle activation, but also with changes in how adjacent joints interacted during the support phase (Madruga-Parera et al., 2025).

The value of inter-joint coordination analysis is that it provides information beyond single-joint kinematics. SAC is not performed through isolated movement of the hip, knee, or ankle; rather, successful execution requires the lower-limb joints to coordinate braking, body support, and propulsion within a very short time. Therefore, changes in coordination patterns may reflect adjustments in movement organization. In the present study, the more evident changes in hip–knee and knee–ankle coordination suggest that the intervention may have influenced joint couplings that are more directly involved in absorbing load and redirecting movement during SAC (Villanueva-Guerrero et al., 2025).

However, the coordination results should also be interpreted carefully. Vector coding provides a descriptive and quantitative representation of joint coupling behavior, but it does not directly reveal the underlying neural control strategy. A change in the proportion of a coordination pattern does not necessarily mean that movement control has improved. It may indicate a more effective strategy, but it may also reflect a compensatory adjustment, a change in movement constraints, or variability caused by the acute intervention. Therefore, the present results should be understood as evidence that the combined intervention altered coordination behavior, rather than definitive evidence that coordination quality was improved.

The joint-specific nature of the findings is also meaningful. The hip–ankle coupling showed fewer significant changes than the hip–knee and knee–ankle couplings. This may indicate that the acute intervention primarily affected adjacent joint relationships that are more directly involved in the SAC support phase. It is also possible that hip–ankle coordination is less sensitive to this type of intervention or that its functional role is more indirect during the specific cutting task used in this study (Gonzalo-Skok and Bishop, 2023). Thus, the coordination response appears to be task- and coupling-specific rather than a global improvement across the whole lower limb.

Because this study did not include direct neurophysiological measures, the mechanisms underlying the coordination changes remain inferential. Neural indices such as the H-reflex, V-wave, V/Mmax ratio, and TMS-based corticospinal excitability could provide additional information about spinal excitability, efferent neural drive, and corticospinal modulation. Without these measures, it is not possible to determine whether the observed coordination changes were driven by spinal-level modulation, supraspinal excitability, peripheral muscle activation, or changes in task execution strategy. Therefore, the coordination findings should be viewed as biomechanical evidence of altered movement organization, not direct evidence of neural adaptation. The main contribution of this study is that it links an acute performance response to both muscle activation and inter-joint coordination during a basketball-relevant SAC task. Previous research has often focused on whether conditioning activities improve subsequent performance, but performance outcomes alone cannot explain how the movement is reorganized after the intervention. By combining V-cut performance, EMG amplitude variables, and vector-coded joint coordination, the present study provides a more comprehensive description of the acute response to NMES combined with weighted squats (Jimenez-Iglesias et al., 2025).

However, the evidential boundary of the study must be clearly stated. The present data show associations among the combined intervention, improved V-cut performance, increased amplitude-based EMG variables in selected muscles, and altered joint coordination patterns. These associations support the possibility that NMES combined with weighted squats acutely changes the neuromuscular and biomechanical state of the athlete before SAC-related performance. However, they do not establish the exact physiological pathway responsible for the performance change.

### 4.4 Limitations and future directions

This study has several limitations. First, all participants were experienced male basketball players, which may limit the generalizability of the findings to female athletes, athletes from other sports, or less experienced individuals. Second, although the protocol included a moderate load, low repetition volume, rest intervals, and a 6-min recovery period to reduce excessive fatigue, fatigue was not directly quantified. The EMG analysis focused on RMS and iEMG, which reflect activation-related changes rather than fatigue-specific indicators. Third, this study examined only acute responses after a single intervention session; therefore, the findings should not be interpreted as evidence of long-term training adaptation. Finally, alternative explanations such as task familiarity, expectancy effects, acute arousal, or changes in movement strategy cannot be completely excluded.

Future studies should include broader participant populations, direct fatigue-related measurements, and longer-term training designs. Frequency-domain EMG variables, such as median frequency and mean frequency, should be included to better evaluate fatigue-related changes. Neurophysiological measures, including the H-reflex, V-wave, V/Mmax ratio, and TMS-based corticospinal excitability, may also help clarify whether the acute response to NMES combined with weighted squats is mainly related to peripheral muscle activation, spinal excitability, corticospinal modulation, or changes in movement strategy. The present study did not perform median-frequency analysis of the EMG signals. Median frequency is mainly used as a frequency-domain indicator of muscle fatigue and changes in EMG spectral characteristics. However, fatigue development was not a primary outcome of the present study. Instead, this study focused on the acute effects of NMES combined with weighted squats on SAC-related performance, muscle activation intensity, and lower-limb joint coordination. Therefore, RMS and iEMG were selected *a priori* as the main EMG variables to reflect amplitude-based neuromuscular activation responses. In addition, because the SAC support phase is brief and highly dynamic, median-frequency estimates within short task-specific windows may have limited stability and interpretability. This issue has been acknowledged as a limitation, and future studies may include frequency-domain EMG analyses to further explore fatigue-related or neuromuscular spectral mechanisms.

## 5 Conclusion

This study showed that NMES combined with weighted squats was more effective than weighted squats alone or sham stimulation combined with weighted squats in acutely improving SAC-related performance. Only the TSLS group demonstrated a significant improvement in V-cut test performance after the intervention. At the same time, this combined intervention produced significant increases in RMS and iEMG mainly in the GM, VM, and BF, and induced more evident changes in lower limb joint coordination, particularly in the hip–knee and knee–ankle couplings. These findings suggest that the acute enhancement in SAC-related performance was accompanied by improved activation of key lower-limb muscles and adjustments in joint coordination strategy. Overall, NMES combined with weighted squats appears to be an effective acute intervention for improving SAC-related performance and movement control.

## Data Availability

The original contributions presented in the study are included in the article/**Supplementary Material**, further inquiries can be directed to the corresponding author/s.
