# Supplementary material for: Sensor-based assessment of the acute effects of NMES combined with weighted squats on muscle activation and lower limb joint coordination during the stop-and-cut task
Source: Front Physiol. 2026 Jul 1;17:1843577. doi: 10.3389/fphys.2026.1843577 (PMC13368641; doi:10.3389/fphys.2026.1843577)
Supplement: Supplementary file 1 [file Table1.docx]

Appendix 1. Analysis of Main Effects of Joint Coordination

| Coordinated Pairs (Pattern) | LS | SSLS |  | TSLS | Pro | Post |
| --- | --- | --- | --- | --- | --- | --- |
| Hip and Knee (Same Phase) | t=-3.93, p=0.006*, d=-1.39 | t=-4.15, p=0.004*, d=-1.47 |  | t=2.25, p=0.059, d=0.79 | F=6.16, p=0.008*, η²=0.37 | F=6.01, p=0.009*, η²=0.36 |
| Hip and Knee (Distal) | t=-0.68, p=0.516, d=-0.24 | t=5.99, p<0.001*, d=2.12 |  | t=-18.23, p<0.001*, d=-6.44 | F=38.96, p<0.001*, η²=0.79 | F=3.70, p=0.042*, η²=0.26 |
| Hip and Ankle (Distal) | t=3.71, p=0.008*, d=1.31 | t=-0.80, p=0.452, d=-0.28 |  | t=5.42, p<0.001*, d=1.92 | F=6.97, p=0.005*, η²=0.40 | F=0.17, p=0.844, η²=0.02 |
| Knee and Ankle (Same Phase) | t=-2.91, p=0.023*, d=-1.03 | t=0.65, p=0.537, d=0.23 |  | t=3.54, p=0.009*, d=1.25 | F=7.74, p=0.003*, η²=0.42 | F=11.71, p<0.001*, η²=0.53 |
| Knee and Ankle (Reverse) | t=-2.80, p=0.026*, d=-0.99 | t=0.75, p=0.476, d=0.27 |  | t=-4.22, p=0.004*, d=-1.49 | F=2.70, p=0.090, η²=0.20 | F=6.96, p=0.005*, η²=0.40 |
| Knee and Ankle (Proximal) | t=-1.45, p=0.190, d=-0.51 | t=3.07, p=0.018*, d=1.08 |  | t=-4.25, p=0.004*, d=-1.50 | F=0.16, p=0.853, η²=0.01 | F=17.21, p<0.001*, η²=0.62 |
| Knee and Ankle (Distal) | t=2.06, p=0.078, d=0.73 | t=0.76, p=0.472, d=0.27 |  | t=6.33, p<0.001*, d=2.24 | F=1.96, p=0.165, η²=0.16 | F=2.34, p=0.121, η²=0.18 |
